# Predicting Obesity in Adults Using Machine Learning Techniques: An Analysis of Indonesian Basic Health Research 2018

**DOI:** 10.3389/fnut.2021.669155

**Published:** 2021-06-21

**Authors:** Sri Astuti Thamrin, Dian Sidik Arsyad, Hedi Kuswanto, Armin Lawi, Sudirman Nasir

**Affiliations:** ^1^Department of Statistics, Faculty of Mathematics and Natural Science, Hasanuddin University, Makassar, Indonesia; ^2^Department of Epidemiology, Faculty of Public Health, Hasanuddin University, Makassar, Indonesia; ^3^Department of Mathematics, Faculty of Mathematics and Natural Sciences, Hasanuddin University, Makassar, Indonesia; ^4^Department of Health Promotion, Faculty of Public Health, Hasanuddin University, Makassar, Indonesia

**Keywords:** classification, Logistic Regression, machine learning, Naive Bayes, obesity status

## Abstract

Obesity is strongly associated with multiple risk factors. It is significantly contributing to an increased risk of chronic disease morbidity and mortality worldwide. There are various challenges to better understand the association between risk factors and the occurrence of obesity. The traditional regression approach limits analysis to a small number of predictors and imposes assumptions of independence and linearity. Machine Learning (ML) methods are an alternative that provide information with a unique approach to the application stage of data analysis on obesity. This study aims to assess the ability of ML methods, namely Logistic Regression, Classification and Regression Trees (CART), and Naïve Bayes to identify the presence of obesity using publicly available health data, using a novel approach with sophisticated ML methods to predict obesity as an attempt to go beyond traditional prediction models, and to compare the performance of three different methods. Meanwhile, the main objective of this study is to establish a set of risk factors for obesity in adults among the available study variables. Furthermore, we address data imbalance using Synthetic Minority Oversampling Technique (SMOTE) to predict obesity status based on risk factors available in the dataset. This study indicates that the Logistic Regression method shows the highest performance. Nevertheless, kappa coefficients show only moderate concordance between predicted and measured obesity. Location, marital status, age groups, education, sweet drinks, fatty/oily foods, grilled foods, preserved foods, seasoning powders, soft/carbonated drinks, alcoholic drinks, mental emotional disorders, diagnosed hypertension, physical activity, smoking, and fruit and vegetables consumptions are significant in predicting obesity status in adults. Identifying these risk factors could inform health authorities in designing or modifying existing policies for better controlling chronic diseases especially in relation to risk factors associated with obesity. Moreover, applying ML methods on publicly available health data, such as Indonesian Basic Health Research (RISKESDAS) is a promising strategy to fill the gap for a more robust understanding of the associations of multiple risk factors in predicting health outcomes.

## Introduction

Obesity is a major health problem strongly associated with many chronic illnesses with negative effects and long-term consequences, not only for the patients but also their families. In Southeast Asia, problems related to nutrition or malnutrition are a double burden because the number of cases of malnutrition and malnourishment is still relatively high and the number of cases of obesity has also increased significantly over time (1).

Data from the 2013 national-level survey of Indonesian Basic Health Research (RISKESDAS) showed the prevalence of obesity in Indonesia has increased over the years. Obesity among adult men was 13.9% in 2007, 7.8% in 2010, and 19.7% in 2013, whereas for adult women the prevalence was 14.8% in 2007, 15.5% in 2010, and increased drastically to 32.9% in 2013 (2). By 2018, the same survey (RISKESDAS 2018) showed that the prevalence of obesity in men and women had decreased slightly to 14.5 and 29.3%, respectively (3).

Risk factors for obesity have been studied extensively, and in general, they are divided into several categories: demographic and socio-economic factors (gender, age, education, income, marital status, and urban areas) (4–6); lifestyle factors (consumption of fast food, stress, smoking, alcoholic drinks, and low level of physical activity) (6, 7); and genetic factors (obese parents) (4, 5). Among these risk factors, some can be changed or modified, while others cannot. Identifying modifiable risk factors for obesity at the individual and the population level is urgently required in order to implement an effective risk reduction strategy. Numerous studies have explored better approaches to predicting obesity using available data. A novel method recently introduced to answer this question uses Machine Learning (ML), which is currently one of the most popular topics in the scientific community for large-scale datasets.

Epidemiological data modeling using ML approaches is becoming increasingly popular in the published scientific literature. These methods have the potential to improve our understanding of general health regarding disease distribution, detection, and the identification of risk factors for health problems, and thus, opportunities for intervention. Various ML methods and algorithms have been applied to various aspects of health data including obesity (8). In the case of obesity, it is essential to develop a precise data classification to facilitate the process of finding predictive risk factors from the given data, in efforts to control these risk factors and eventually to decrease morbidity and mortality linked to obesity.

For the purpose of obesity prevention, ML has been used to predict the probability of obesity based on data encoding adherence to dietary recommendations and several other factors (9). The ML has also been applied for the prediction of obesity in children using electronic health records before the age of 2 (10); prediction of obesogenic environments for children (11); and for the aggregation of metabolomics, lipidomics, and other clinical data to modeling drug dose responses (12).

Based on previous research, ML approaches can increase the risk prediction of health outcomes compared to conventional approaches (13). Prediction of obesity using ML has been investigated by many researchers: Zhang et al. (14), Adnan et al. (15), Toschke et al. (16), Golino et al. (17), Dugan et al. (10), Zheng and Ruggiero (18), Chatterjee et al. (19), Singh and Tawfik (20), and Colmenarejo (21). The ML approach provides an alternative in providing information with a unique approach at the application stage of data analysis on obesity which is important in providing a better predictive solution to the likelihood of obesity (22).

## Materials and Methods

### Data Source

The dataset used to develop the classification model in this study is publicly available data from an Indonesia national scale survey with a cross-sectional and non-intervention design, the RISKESDAS survey, which was conducted by the Indonesian Ministry of Health. The RISKESDAS report is a community-based health survey whose indicators can be generalized with variables described from the national level down to the district/city level. It is conducted every 5 years across 34 provinces and 514 districts/cities in order to track important indicators of public health status, diseases risk factors, and to evaluate healthcare services delivery programs. The methodology and detailed protocols of the survey are described elsewhere (3). Briefly, the target sample for this study is 300,000 households from 30,000 Census Block (CBs) in 34 provinces and 514 district-cities throughout Indonesia. The sampling frame lists are provided by the Central Bureau of Statistics (BPS) using a two-stage sampling method. In the first stage, 180,000 CBs (25%) were selected from 720,000 CBs from the national socio-economic survey (SUSENAS) as a sampling frame using a proportionate to population size (PPS) method and stratified by prosperity level, continued by systematically selecting 30,000 CBs from 180,000 CBs priorly selected and stratified by urban and rural for each district or city. In the second stage, 10 households were selected systematically using implicit stratification for the education level of the head of household to maintain variation of education among households. Household members who were eligible according to the inclusion criteria were invited to participate in the interview.

The dataset can be accessed by request at the Institute of Health Research and Development of the Indonesian Ministry of Health (https://www.litbang.kemkes.go.id/layanan-permintaan-data-riset/).

### Pre-processing Data

#### Data Cleaning or Filtering

The sample used in this study included all the data from the RISKESDAS dataset for individuals aged 18 or above; in total there was data for 634,709 respondents. We conducted data cleaning by excluding all records with incomplete or missing values for the variable/feature Body Mass Index (BMI), a core feature used to categorize obesity status. The number of samples included for the analysis process after cleaning was 618,898 records. Data cleaning was performed by using the *dplyr* package of R version 3.5.1 to perform filtering (23).

#### Feature Selection

After removing missing values, we proceeded to variable or feature selection. Variable selection is a process of reducing the data dimensions to reduce processing time as well as computation costs (24). We selected a subset of variables that contributed significantly to the target class to improve the overall predictive performance of the classification using the Chi-Square (χ^2^) test between obesity status with each of the variables and including those with a *p*-value <0.05. All features that met these criteria (a total of 21 features) were selected for developing the classification model. These variables or features were location (X1), marital status (X2), age group (X3), education (X4), work category (X5), sugary foods (X6), sweet drinks (X7), salty foods (X8), fatty/oily foods (X9), grilled foods (X10), preserved foods (X11), seasoning powders (X12), soft/carbonated drinks (X13), energy drinks (X14), instant foods (X15), alcoholic drinks (X16), mental-emotional disorders (X17), diagnosed hypertension (X18), physical activity (X19), smoking (X20), and fruit and vegetables consumptions (X21). A list of these features and how it was generated from the questionnaire (for composited and calculated feature, i.e., obesity, fruit and vegetables consumption, physical activity, and mental-emotional disorders) can be found in the Supplementary Table 1. The process of developing a classification model was carried out by using the R Statistical Software version 3.5.1 (25).

### Dealing With Imbalanced Datasets

Data imbalance occurs when there are one or more classes that dominate the whole data as major classes, and other classes are rare occurrences or minor classes. Imbalanced data will produce a good classification prediction accuracy against the major class, but in the minor class, the resulting accuracy is poor.

The Synthetic Minority Oversampling Technique (SMOTE) was introduced by Chawla et al. (26) and Chawla (27), as a way of dealing with the effect of the lack of information on minority classes in a data set. SMOTE is an algorithm with an oversampling approach, which generates artificial data for minority data classes (28) so that the proportions of major and minor data classes are more balanced (29). Artificial data or synthetic data are made based on the *k*-nearest neighbor. All attributes used in this study were categorical features so that the calculation of the distance between the minor class samples was carried out using the Modify Value Difference Metric (MVDM) method (30). In this method, several steps are taken, namely calculating the distance between two observations at a nominal scale and choosing the majority category between the minority class observations with its *k*-closest neighbors for a nominal value, and if the same value occurs, it is chosen randomly. Furthermore, the selected value is a new observation. In this study, the SMOTE technique with oversampling of 200% and 300% was used which resulted in two new datasets.

### Machine Learning Classification Methods

#### Logistic Regression

One of the basic linear models developed with a probabilistic approach to classification problems is Logistic Regression (31) and is one of the supervised learning models widely used in ML. Logistic Regression can be seen as a development of Linear Regression models with a logistic function for data with a target in the form of classes (32) as follows:

$$\begin{array}{l} y\left(x\right)=\sigma \left(\beta_{0}+\beta^{T}x\;\right), \end{array}$$

where $x={\left(x_{1},x_{2},\ldots ,x_{D}\right)}^{T}$ is the *D-*dimensional data, $\beta ={\left(\beta_{1},\beta_{2},\ldots ,\beta_{D}\right)}^{T}$ are the weight parameters, β_0_ is the bias parameter, and σ is a logistic function that is shaped as $\sigma \left(a\right)=\frac{1}{1+e^{-a}}$.

The weights of β can be obtained by using probabilistic concepts. For example, if *y*_*n*_ = *y*(*x*_*n*_) and *t*_*n*_ ∈ {0, 1} are an independent identical distribution. The joint probabilistic or likelihood function for all the data can be expressed by the Bernoulli distribution *p*(*t*|β), where $t={\left(t_{1},t_{2},\ldots ,t_{N}\right)}^{T}$. Therefore, the Logistic Regression learning and bias (β) is to maximize *p*(*t*∨β). The learning method for determining the weight and bias (β) parameters is known as the maximum likelihood method. Generally, the solution to the maximum likelihood problem is done by minimizing the negative of the logarithm of the likelihood function, namely $\underset{\beta }{\min}\;E\left(\beta \right)$, where *E*(β) = −ln(*p*(*t*∨β)). Logistic Regression models can use regularization techniques to solve the problem of overfitting by adding the weight norm ||w|| in the error function, namely $E\left(\beta \right)=\frac{1}{2}||\beta |{|}^{2}+C\overset{N}{\underset{n=1}{\sum }}\left\{t_{n}\ln\left(y_{n}\right)+\left(1-t_{n}\right)\ln\left(1-y_{n}\right)\right\}$, where C > 0 is the inverse parameter of the regulation.

Simultaneous and partial parameter testing is performed to examine the role of predictor variables in the model. Simultaneous parameter testing uses the G test.

#### Classification and Regression Trees

Breiman et al. (33) proposes a new algorithm for tree arrangement, namely Classification and Regression Tree (CART). CART is a non-parametric statistical method used for classification analysis, both for categorical and continuous response variables, and for explanatory variables which may consist of nominal, ordinal, or continuous features. The resulting tree model depends on the scale of the response attribute. CART generates a classification tree if the response variables are categorical, and generates a regression tree if the response variables are continuous (33).

The tree structure in the CART method is obtained through a binary recursive partitioning algorithm against its explanatory variables (31, 32). The binding is carried out by dividing the data set into two subclusters called nodes. The impurity value at node *t* is a measurement of the heterogeneity level of a class from a particular node in the classification tree. The process of forming a classification tree is carried out in three stages; selecting a classifier, determining the final node, and marking the class label (31). In selecting the classifier, each partitioning depends on the value that comes from only one explanatory variable. For categorical variables, the partitioning that occurs comes from all the possible partitioning based on the formation of two subgroups that are mutually exclusive (disjoint). In addition, in solving classification tree problems, the Gini Splitting Rule (also known as the Gini Index) is the most common rule to be used (32). Then, the partitioning evaluation is performed using the goodness of split φ(*s, t*) of the *s* partition at *t* node. The partitioning function is defined as decreased heterogeneity. A sort that produces a higher value is a better sort because it reduces the impurity value more significantly. If the resulting node is of a non-homogeneous class, the same procedure will be repeated until the tree $\varphi \left(s,t\right)\varphi \left(s^{*},t\right)=\underset{s\in S}{\max}\varphi \left(s,t\right)$. Determination of child nodes is carried out recursively by using the same method as determining the main node.

After selecting the classifier, the end node is determined. The minimum number of cases in a node is generally five. If this is fulfilled, tree development will be stopped and continued with the marking of class labels. Class label marking at the end node is carried out based on the highest number rule. The process of forming classification trees stops when there is only one observation in each child node. One of the ways to get the optimal tree is by consecutively pruning the tree that is less important. In random pruning, the observations are divided into two parts, namely training data *L*_1_ and test data *L*_2_. Through the pruning process, a row of trees is formed from *L*_1_. Next, *L*_2_ is used to form the total proportion of misclassification (*R*|*ts*(*G*)). The optimal tree that meets the criteria as $R^{ts}\left(G^{0}\right)=\min{\;R}^{ts}\left(G_{t}\;\right)$.

#### Naïve Bayesian

Naïve Bayesian classification is a statistical approach which attempts to predict the probability of each class (14). The advantage of this Bayes grouping is that it has a high level of accuracy and speed when using large data sets. Naïve Bayesian grouping assumes that the values of the variables on the class labels are independent of other attribute values, which can facilitate the calculation (10, 34).

Naïve Bayesian Classification is achieved by applying the Bayes rule to calculate the probability of each attribute and predicting the class based on the highest prior probability (34).

### Model Validation

The validation process in this study used *k*-fold cross-validation (35). Cross-Validation (CV) divides the dataset into two parts: one part is used as the training data and the other is used as testing data. In this study, the data were divided into 10 parts, 90% of which was used as training and the rest was used for testing. This process was done repeatedly, a maximum of 10 times, until all data records were part of the testing data. This process is also known as the 10-fold CV. The 10-fold CV process has been used in several previous health care- and medical-related studies (36).

### Evaluation of Classification Performance

Measuring accuracy is a diagnostic step to test the level of performance of an algorithm against the dataset used. A matrix, known as the confusion matrix, is used to evaluate the learning algorithm (37). Each column in the matrix shows the number of observations in the predicted class. The rows in the matrix represent the actual number of observations in the class.

In ML, the term metric refers to a value that can be used to represent the performance of the resulting model. In classification modeling, the model output is a label/class. There are several metrics that are commonly used, namely accuracy, precision, sensitivity, specificity, recall, F1-score, kappa, and *F*_β_. In terms of the confusion matrix, accuracy is the ratio of the number of diagonal elements to the total number of matrix elements. The accuracy of the method is only considered adequate when the comparison of the actual number of data labels is nearly identical with the confusion matrix. If the comparison is imbalanced, then other metrics can be used. Precision is an appropriate metric when false positives are to be avoided. Sensitivity can be interpreted as the degree of reliability of the model to detect data labeled positive correctly. Sensitivity is an appropriate metric when false negatives are to be avoided (high risk). Specificity is the degree of model reliability for detecting data labeled negative correctly. This metric is closely related to sensitivity. This metric is appropriate when the true negative rate is to be maximized. To minimize both (false positive and false negative) outcomes at the same time, precision and sensitivity need to be summarized by using the F1-score. Recall is a valid choice of evaluation metric when we want to capture as many positives (obese) as possible. In this study, we want to be sure that the sample we catch is obese (precision) and we also want to capture as many obese (recall) as possible. The F1-score manages this trade-off. However, the main problem with the F1-score is that it gives equal weight to precision and recall. Sometimes we may need to include domain knowledge in our evaluations where we want more recall or more precision. To solve this, we can create a weighted F1 metric, where beta (β) sets the balance between precision and recall. This is called *F*_β_. In this study, we used β = 0.5 to measure more weight on precision and less weight on recall.

Kappa is used to test the inter reliability. Kappa values range from 0 to 1.0 which can be divided into several classifications, namely 0–0.20 (slight), 0.21–0.40 (fair), 0.41–0.60 (moderate), 0.61–0.80 (substantial), and 0.81–1.0 (perfect) (38).

The Area Under ROC Curve, also known as AUC, has a range between 0.5 (50%) and 1 (100%). The interpretation of AUC values can be classified into five different sections, namely 0.5–0.6 (false accuracy), 0.6–0.7 (poor accuracy), 0.7–0.8 (moderate accuracy), 0.8–0.9 (high accuracy), and 0.9–1 (very high level of accuracy) (39).

## Results

An overview of the explanatory variables contained in the obesity data of the Indonesia RISKESDAS 2018 survey is given in Table 1. As can be seen from Table 1, out of 618,898 respondents, there are 134,709 (21.77%) people who are classified as obese, 484,189 (78.23%) people are non-obese. In Table 1, it can also be seen that the number of obese (21.77%) and non-obese classes (78.23%) seems imbalanced. Based on Table 1, the respondents in this study lived in rural areas (56.71%), married (76.31%), aged 35–39 years (12.53%), finished senior high school (25.43%), unemployed (27.79%), consumed sugary foods 1–2 times per week (28.63%), drank sweet drinks one time per day (31.57%), consumed salty foods 1–2 times per week (27.54%), consumed fatty/oily foods 1–2 times per week (26.61%), consumed grilled foods more than 3 times per month (32.68%), never consumed preserved foods (56.70%), consumed seasoning powders less that one time per day (36.74%), never drank soft/carbonated drinks (72.19%), never drank energy drinks (81.58%), experienced no mental emotional disorders (90.13%), consumed instant foods 1–2 times per week (35.57%), drank non-alcoholic drinks (95.11%), diagnosed with no hypertension (50.97%), not adequate physical activity (88.09%), not a smoker (62.30%), and consumed inadequate fruit and vegetables (95.26%). This general description of the obesity data can be seen in detail in Table 1. Moreover, the obesity status description can be seen in detail in the Supplementary Table 2.

**Table 1:** General description of obesity data from Indonesian RISKESDAS 2018.

**Variables**	**Categories**	**Frequency**	**Percentage**
Obesity status (Y)	Non-obese	484,189	78.23
	Obese	134,709	21.77
Location (X1)	Urban	267,913	43.29
	Rural	350,985	56.71
Marital status (X2)	Not married	84,792	13.70
	Married	472,269	76.31
	Divorced	14,333	2.32
	Widowed	47,504	7.68
Age groups (X3)	18–24 years	69,532	11.23
	25–29 years	60,380	9.76
	30–34 years	68,683	11.10
	35–39 years	77,538	12.53
	40–44 years	73,775	11.92
	45–49 years	70,503	11.39
	50–54 years	58,618	9.47
	55–59 years	49,632	8.02
	60–64 years	35,471	5.73
	>64 years	54,766	8.85
Education (X4)	Not/Never schooled	40,861	6.60
	Not finished basic school	84,637	13.68
	Finished basic school	157,391	25.43
	Finished Junior High School	104,435	16.87
	Finished Senior High School	170,246	27.51
	Finished Academy/College	20,005	3.23
	Finished higher education	41,323	6.68
Work types (X5)	Not working	171,984	27.79
	School	12,238	1.98
	Government employee	27,703	4.48
	Private employee	50,049	8.09
	Entrepreneur	91,011	14.71
	Farmer	163,009	26.34
	Fisherman	8,344	1.35
	Daily waged labors	52,379	8.46
	Others	42,181	6.82
Sugary foods (X6)	>1 time per day	82,775	13.37
	1 time per day	125,754	20.32
	3–6 times per week	138,685	22.41
	1–2 times per week	177,173	28.63
	<3 times per month	62,972	10.17
	Never	31,539	5.10
Sweet drinks (X7)	>1 time per day	176,096	28.45
	1 time per day	195,361	31.57
	3–6 times per week	87,827	14.19
	1–2 times per week	95,409	15.42
	<3 times per month	33,666	5.44
	Never	30,539	4.93
Salty foods (X8)	>1 time per day	64,660	10.45
	1 time per day	78,744	12.72
	3–6 times per week	105,363	17.02
	1–2 times per week	170,442	27.54
	<3 times per month	107,318	17.34
	Never	92,371	14.93
Fatty/Oily foods (X9)	>1 time per day	103,634	16.74
	1 time per day	113,057	18.27
	3–6 times per week	133,552	21.58
	1–2 times per week	164,703	26.61
	<3 times per month	72,739	11.75
	Never	31,213	5.04
Grilled foods (X10)	>1 time per day	12,948	2.09
	1 time per day	22,189	3.59
	3–6 times per week	63,967	10.34
	1–2 times per week	161,356	26.07
	<3 times per month	202,251	32.68
	Never	156,187	25.24
Preserved foods (X11)	>1 time per day	6,310	1.02
	1 time per day	12,024	1.94
	3–6 times per week	31,993	5.17
	1–2 times per week	72,618	11.73
	<3 times per month	145,068	23.44
	Never	350,885	56.70
Seasonings powders (X12)	>1 time per day	227,357	36.74
	1 time per day	226,628	36.62
	3–6 times per week	42,598	6.88
	1–2 times per week	34,030	5.50
	<3 times per month	20,887	3.37
	Never	67,398	10.89
Soft/Carbonated drinks (X13)	>1 time per day	3,689	0.60
	1 time per day	7,857	1.27
	3–6 times per week	16,470	2.66
	1–2 times per week	43,686	7.06
	<3 times per month	100,398	16.22
	Never	446,798	72.19
Energy drinks (X14)	>1 time per day	3,654	0.59
	1 time per day	7,761	1.25
	3–6 times per week	12,888	2.08
	1–2 times per week	31,045	5.02
	<3 times per month	58,659	9.48
	Never	504,891	81.58
Instant foods (X15)	>1 time per day	12,144	1.96
	1 time per day	28,943	4.68
	3–6 times per week	108,287	17.50
	1–2 times per week	220,125	35.57
	<3 times per month	149,066	24.09
	Never	100,333	16.21
Alcoholic drinks (X16)	Yes	30,240	4.89
	No	588,658	95.11
Mental-emotional disorders (X17)	Yes	61,092	9.87
	No	557,806	90.13
Diagnosed hypertension (X18)	Yes	55,640	8.99
	No	315,467	50.97
	Unknown	247,791	40.04
Physical activity (X19)	Adequate	73,736	11.91
	Not adequate	545,162	88.09
Smoking (X20)	Yes	233,306	37.70
	No	385,592	62.30
Fruit and vegetables consumptions (X21)	Adequate	29,321	4.74
	Not adequate	589,577	95.26

To overcome the oversampling of the prediction of this obesity status classification due to class imbalance in the dataset (Table 1), the SMOTE technique was used. In this study, the SMOTE technique used two different percentages, namely 200% and 300%. SMOTE with 300% can improve minor class data better (from 21.77%, in the original dataset, to 47.3%). As a result, the comparison between major class (non-obese) and minor class (obese) is balanced, namely 47.3% and 52.7%, respectively. The new dataset resulting from the SMOTE technique with 300% was used to build a classification model and prediction of obesity risk factors.

Using the three models (Logistic Regression model, CART, and Naïve Bayes), 10-fold CV was carried out to train and see which model performed better in predicting test set points on all data (Tables 2, 3). This is also to ensure that all these new data resulting from the SMOTE technique are not bias in the result.

**Table 2:** Comparison of classification accuracy with 10-fold CV based on the obesity test data using three models with confusion matrix.

**ML methods**	**Classification prediction**	**Fold 1 Test**		**Fold 2 Test**		**Fold 3 Test**		**Fold 4 Test**		**Fold 5 Test**	
		**Real circumstances**									
		**Non-obese**	**Obese**	**Non-obese**	**Obese**	**Non-obese**	**Obese**	**Non-obese**	**Obese**	**Non-obese**	**Obese**
CART	Non-obese	360,554	193,472	360,260	193,579	360,791	193,595	360,325	193,504	360,459	193,685
	Obese	75,298	291,411	75,283	291,744	75,227	291,362	75,294	291,335	75,401	291,611
Naïve-Bayes	Non-obese	314,384	141,264	313,957	141,209	314,357	141,167	314,080	141,106	314,273	141,413
	Obese	121,468	343,619	121,586	344,114	121,661	343,790	121,539	343,733	121,587	343,883
Logistic Regression	Non-obese	320,456	140,260	319,952	140,279	320,628	140,336	320,202	140,144	320,285	140,474
	Obese	115,396	344,623	115,591	345,044	115,390	344,621	115,417	344,695	115,575	344,822
**ML methods**	**Classification prediction**	**Fold 6 Test**		**Fold 7 Test**		**Fold 8 Test**		**Fold 9 Test**		**Fold 10 Test**	
		**Real circumstances**									
		**Non-obese**	**Obese**	**Non-obese**	**Obese**	**Non-obese**	**Obese**	**Non-obese**	**Obese**	**Non-obese**	**Obese**
CART	Non-obese	360,531	193,271	360,426	193,360	360,177	193,275	360,566	193,586	360,411	193,430
	Obese	75,312	291,645	75,410	291,447	75,351	291,331	75,308	291,504	75,317	291,377
Naïve-Bayes	Non-obese	314,356	141,221	314,273	141,183	314,030	141,113	314,239	141,296	314,234	141,345
	Obese	121,487	343,695	121,563	343,624	121,498	343,493	121,635	343,794	121,494	343,462
Logistic Regression	Non-obese	320,479	140,281	320,423	140,220	320,206	140,253	320,464	140,277	320,355	140,328
	Obese	115,364	344,635	115,413	344,587	115,322	344,353	115,410	344,813	115,373	344,479

**Table 3:** Evaluation of classification prediction performance with 10-fold CV based on the obesity test data using 3 ML methods.

**ML methods**	**Test**	**Accuracy (%)**	**Sensitivity (%)**	**Specificity (%)**	**Precision (%)**	**F1-Score (%)**	**Kappa (%)**	**AUC (%)**	***F_**β**_*_** = 0.5**_ (%)**
CART	1-Fold	70.81	**82.72**	60.10	65.08	**72.85**	42.24	74.57	67.98
	2-Fold	70.80	**82.72**	60.11	65.05	**72.83**	42.24	74.56	67.95
	3-Fold	70.81	**82.75**	60.08	65.08	**72.86**	42.25	74.56	67.98
	4-Fold	70.80	**82.72**	60.09	65.06	**72.83**	42.22	74.55	67.96
	5-Fold	70.79	**82.70**	60.09	65.05	**72.82**	42.21	74.54	67.95
	6-Fold	70.83	**82.72**	60.14	65.10	**72.86**	42.28	74.55	68.00
	7-Fold	70.81	**82.70**	60.12	65.08	**72.84**	42.24	74.55	67.98
	8-Fold	70.81	**82.70**	60.12	65.08	**72.84**	42.24	74.56	67.97
	9-Fold	70.80	**82.72**	60.09	65.07	**72.84**	42.23	74.56	67.97
	10-Fold	70.81	**82.71**	60.10	65.07	**72.84**	42.24	74.54	67.97
Naïve-Bayes	1-Fold	71.46	72.13	70.87	69.00	70.53	42.90	78.47	69.60
	2-Fold	71.46	72.08	70.90	68.98	70.50	42.89	78.47	69.58
	3-Fold	71.46	72.10	70.89	69.01	70.52	42.89	78.47	69.61
	4-Fold	71.47	72.10	70.90	69.00	70.52	42.90	78.47	69.60
	5-Fold	71.45	72.10	70.86	68.97	70.50	42.87	78.45	69.57
	6-Fold	71.47	72.13	70.88	69.00	70.53	42.90	78.48	69.60
	7-Fold	71.46	72.11	70.88	69.00	70.52	42.89	78.46	69.60
	8-Fold	71.46	72.10	70.88	69.00	70.52	42.89	78.45	69.60
	9-Fold	71.45	72.09	70.87	68.98	70.50	42.87	78.48	69.58
	10-Fold	71.45	72.12	70.85	68.97	70.51	42.86	78.47	69.58
Logistic Regression	1-Fold	**72.23**	73.52	**71.07**	**69.56**	71.49	**44.47**	**79.80**	**70.32**
	2-Fold	**72.21**	73.46	**71.10**	**69.52**	71.44	**44.43**	**79.79**	**70.27**
	3-Fold	**72.23**	73.54	**71.06**	**69.56**	71.49	**44.47**	**79.80**	**70.32**
	4-Fold	**72.24**	73.51	**71.09**	**69.56**	71.48	**44.47**	**79.80**	**70.31**
	5-Fold	**72.20**	73.48	**71.05**	**69.51**	71.44	**44.41**	**79.77**	**70.27**
	6-Fold	**72.24**	73.53	**71.07**	**69.55**	71.49	**44.47**	**79.80**	**70.31**
	7-Fold	**72.23**	73.52	**71.08**	**69.56**	71.48	**44.47**	**79.78**	**70.32**
	8-Fold	**72.22**	73.52	**71.06**	**69.54**	71.48	**44.45**	**79.78**	**70.30**
	9-Fold	**72.24**	73.52	**71.08**	**69.55**	71.48	**44.48**	**79.81**	**70.31**
	10-Fold	**72.22**	73.52	**71.05**	**69.54**	71.48	**44.45**	**79.79**	**70.30**

*Bold values shows in which aspect does the ML methods performed best*.

The prediction performance for the classification of obesity status from these methods is also assessed based on accuracy, sensitivity, specificity, precision, recall, F1-score, kappa, and *F*_β_. The measurement results of these metrics based on the 10-fold CV using ML methods for the obesity data set can be seen in Table 3. Based on Table 3, the classification prediction using the Logistic Regression method achieves the best performance based on the accuracy metric (72%), specificity (71%), precision (69%), Kappa (44%), and *F*_β_ (70%). Classification prediction by the CART method achieves the highest sensitivity (82%) and the highest F1-score (72%).

**Figure 1:**
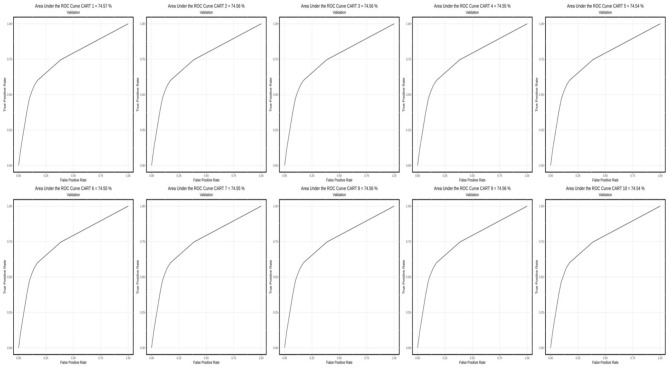
AUC performance of the classification methods with 10-fold CV using the CART method.

**Figure 2:**
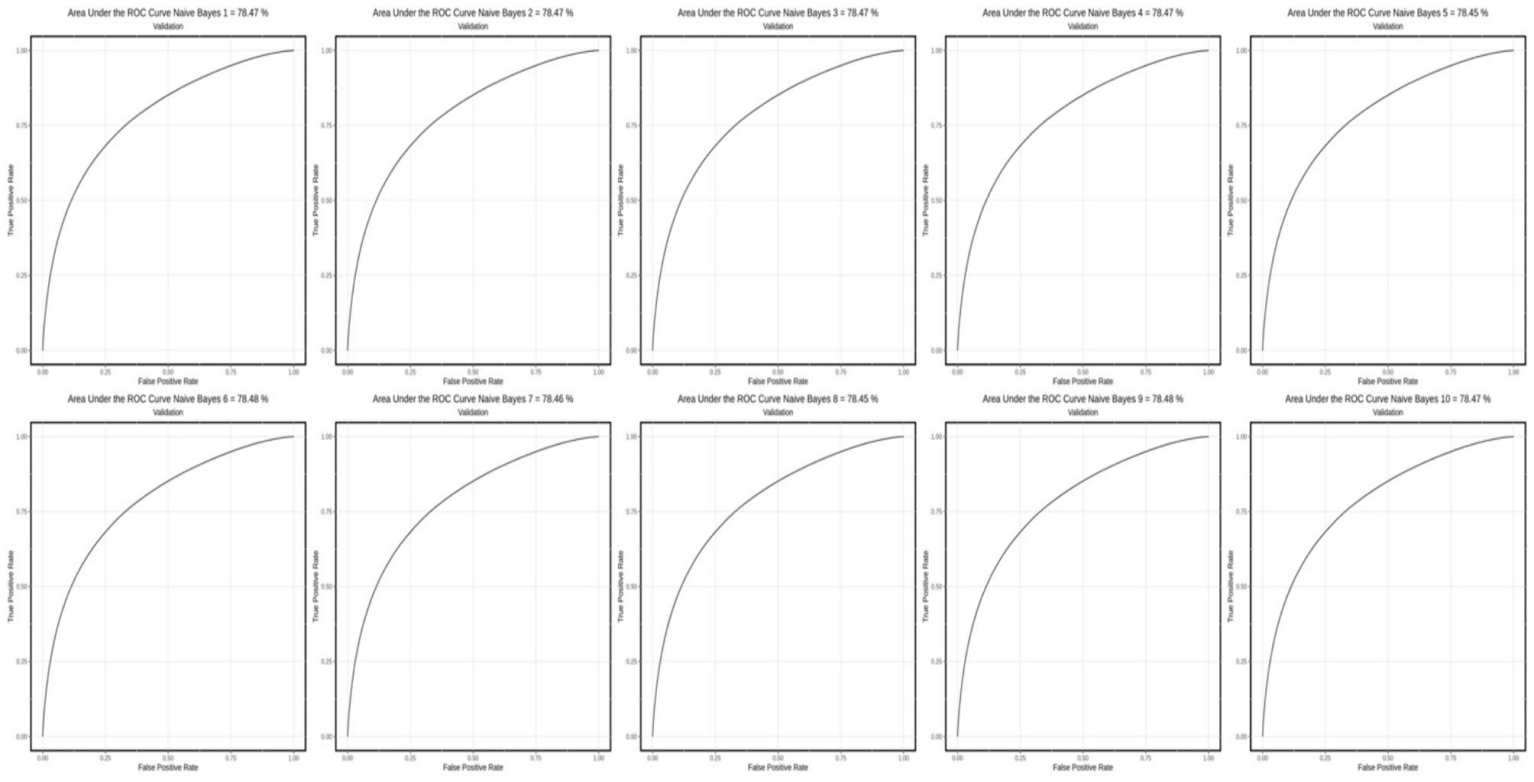
AUC performance on the classification method with the 10-fold CV using the Naïve Bayes method.

**Figure 3:**
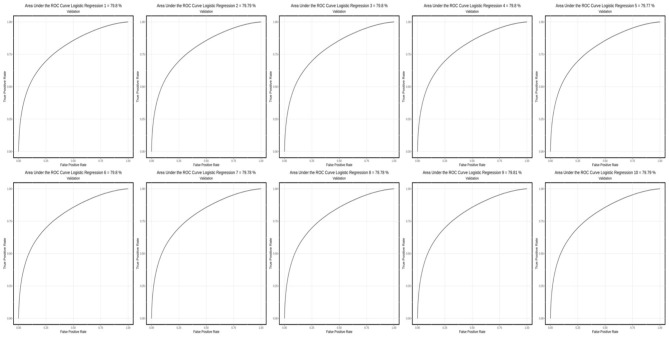
AUC performance on the classification method with the 10-fold CV using the Logistic Regression method.

Figures 1–3 show AUC performance of the respective classification methods with 10-fold CV. The results show that the Logistic Regression classifier has the highest average AUC values (0.798) (Figure 3). In addition to comparing the AUC values obtained, the accuracy, sensitivity, specificity, precision, F1-Score, and *F*_β_ values of each method can also be considered. The AUC is a classification threshold invariant metric that measures the predictive quality of a model regardless of which classification threshold is selected.

After calculating the classification performance for correctly determining the obesity status for each of the 3 different models, it is also necessary to estimate a set of risk factors for obesity among the available study variables. Based on the evaluation of classification prediction performance, the Logistic Regression method had the better performance compared with the CART method and the Naïve Bayes method. Overall, fold 6 out of 10-fold CV showed the best accuracy for the classification performance of the obesity status. Partial testing of parameters of the Logistic Regression model using the Wald test showed that all explanatory variables qualify as factors that can affect the obesity status (Table 4). From Table 4, the variables that have the greatest effect on the obesity status in adults (*p*-value <0.05) included location (X1), marital status (X2), age groups (X3), education (X4), sweet drinks (X7), fatty/oily foods (X9), grilled foods (X10), preserved foods (X11), seasoning powders (X12), soft/carbonated drinks (X13), alcoholic drinks (X16), mental emotional disorders (X17), diagnosed hypertension (X18), physical activity (X19), smoking (X20), and fruit and vegetables consumptions (X21).

**Table 4:** Estimation of the Logistic Regression parameters based on fold 6 out of the 10-fold CV for obesity dataset in Indonesian RISKESDAS 2018 survey.

**Descriptive of variables**		**Fold 6 out of 10-fold CV Test**				
		**β**	**SE**	**Wald**	***p*-Value**	**Odd Ratio**
Constant		6.510	0.046	142.754	0.000	671.976
Location (X1)	Rural	−0.305	0.005	−59.121	0.000	0.737
Marital status (X2)	Married	−0.363	0.007	−50.033	0.000	0.695
	Divorced	0.271	0.015	18.000	0.000	1.311
	Widowed	0.289	0.012	24.963	0.000	1.335
Age groups (X3)	25–29 years	0.488	0.010	46.674	0.000	1.630
	30–34 years	0.560	0.011	52.679	0.000	1.750
	35–39 years	0.680	0.011	64.375	0.000	1.975
	40–44 years	0.746	0.011	69.255	0.000	2.110
	45–49 years	0.741	0.011	67.743	0.000	2.097
	50–54 years	0.549	0.012	46.783	0.000	1.731
	55–59 years	0.333	0.013	26.349	0.000	1.396
	60–64 years	0.304	0.014	21.859	0.000	1.355
	>64 years	−0.457	0.014	−32.580	0.000	0.633
Education (X4)	Not finished basic school	0.313	0.013	24.156	0.000	1.367
	Finished basic school	0.361	0.012	29.692	0.000	1.435
	Finished Junior High School	0.456	0.013	35.808	0.000	1.577
	Finished Senior High School	0.469	0.012	38.083	0.000	1.598
	Finished Academy/College	0.502	0.018	28.496	0.000	1.652
	Finished higher education	0.506	0.015	33.432	0.000	1.659
Work types (X5)	School	−0.356	0.018	−19.850	0.000	0.700
	Government employee	0.197	0.013	15.224	0.000	1.218
	Private employee	−0.117	0.010	−12.055	0.000	0.889
	Entrepreneur	0.069	0.008	8.797	0.000	1.072
	Farmer	−0.548	0.007	−74.090	0.000	0.578
	Fisherman	−0.838	0.024	−35.437	0.000	0.432
	Daily waged labors	−0.389	0.010	−39.463	0.000	0.678
	Others	0.010	0.010	0.987	0.324	1.010
Sugary foods (X6)	1 times per day	−0.135	0.009	−15.096	0.000	0.874
	3–6 times per week	−0.141	0.009	−15.938	0.000	0.869
	1–2 times per week	−0.158	0.009	−18.457	0.000	0.854
	<3 times per month	0.013	0.011	1.189	0.234	1.013
	Never	−0.101	0.014	−7.308	0.000	0.904
Sweet drinks (X7)	1 times per day	0.094	0.007	13.815	0.000	1.099
	3–6 times per week	0.148	0.008	17.454	0.000	1.159
	1–2 times per week	0.189	0.008	22.735	0.000	1.208
	<3 times per month	0.313	0.012	26.572	0.000	1.368
	Never	0.297	0.013	23.106	0.000	1.346
Salty foods (X8)	1 times per day	0.070	0.010	6.824	0.000	1.073
	3–6 times per week	−0.077	0.010	−7.773	0.000	0.926
	1–2 times per week	−0.113	0.009	−12.268	0.000	0.893
	<3 times per month	−0.056	0.010	−5.640	0.000	0.946
	Never	−0.016	0.010	−1.568	0.117	0.984
Fatty/Oily foods (X9)	1 times per day	−0.092	0.009	−10.707	0.000	0.913
	3–6 times per week	−0.158	0.008	−19.229	0.000	0.854
	1–2 times per week	−0.165	0.008	−20.722	0.000	0.848
	<3 times per month	−0.184	0.010	−18.937	0.000	0.832
	Never	−0.495	0.014	−35.457	0.000	0.609
Grilled foods (X10)	1 times per day	−0.184	0.019	−9.749	0.000	0.832
	3–6 times per week	−0.311	0.016	−18.881	0.000	0.733
	1–2 times per week	−0.419	0.016	−26.825	0.000	0.658
	<3 times per month	−0.430	0.016	−27.690	0.000	0.651
	Never	−0.452	0.016	−28.697	0.000	0.636
Preserved foods (X11)	1 times per day	−0.465	0.025	−18.674	0.000	0.628
	3–6 times per week	−0.550	0.022	−25.115	0.000	0.577
	1–2 times per week	−0.597	0.021	−28.800	0.000	0.551
	<3 times per month	−0.694	0.020	−34.273	0.000	0.499
	Never	−0.856	0.020	−42.964	0.000	0.425
Seasonings powders (X12)	1 times per day	0.117	0.006	19.308	0.000	1.124
	3–6 times per week	0.276	0.010	27.709	0.000	1.318
	1–2 times per week	0.229	0.011	20.837	0.000	1.257
	<3 times per month	0.582	0.013	46.073	0.000	1.789
	Never	0.399	0.008	47.027	0.000	1.491
Soft/Carbonated drinks (X13)	1 times per day	0.313	0.032	9.805	0.000	1.368
	3–6 times per week	0.156	0.029	5.284	0.000	1.169
	1–2 times per week	0.073	0.028	2.621	0.009	1.076
	<3 times per month	−0.158	0.027	−5.753	0.000	0.854
	Never	−0.457	0.027	−16.900	0.000	0.633
Energy drinks (X14)	1 times per day	0.046	0.031	1.476	0.140	1.047
	3–6 times per week	0.020	0.029	0.681	0.496	1.020
	1–2 times per week	−0.032	0.027	−1.185	0.236	0.968
	<3 times per month	−0.095	0.027	−3.549	0.000	0.909
	Never	−0.713	0.026	−27.394	0.000	0.490
Instant foods (X15)	1 times per day	0.010	0.019	0.512	0.609	1.010
	3–6 times per week	0.048	0.017	2.767	0.006	1.049
	1–2 times per week	−0.063	0.017	−3.710	0.000	0.939
	<3 times per month	0.084	0.017	4.901	0.000	1.088
	Never	−0.009	0.018	−0.533	0.594	0.991
Alcoholic drinks (X16)	No	−1.576	0.008	−190.048	0.000	0.207
Mental-emotional disorders (X17)	No	−1.029	0.007	−150.755	0.000	0.357
Diagnosed hypertension (X18)	No	−0.867	0.009	−100.728	0.000	0.420
	Unknown	−0.982	0.009	−110.600	0.000	0.375
Physical activity (X19)	Not adequate	−0.852	0.007	−128.275	0.000	0.427
Smoking (X20)	No	0.219	0.005	41.165	0.000	1.244
Fruit and vegetables consumptions (X21)	Not adequate	−1.248	0.009	−135.504	0.000	0.287

**Figure 4:**
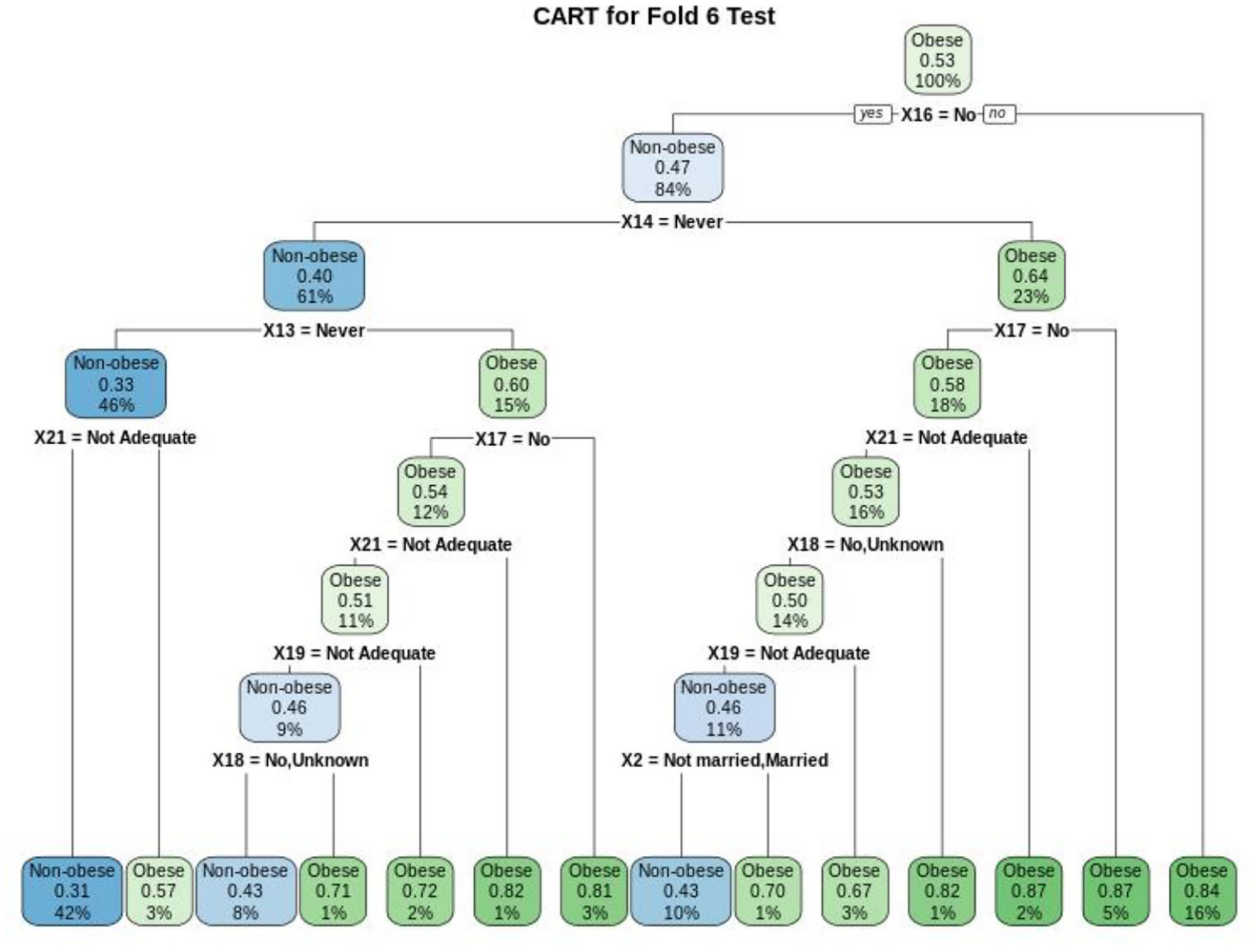
Obesity data classification tree for fold 6 out of the 10-fold CV for CART model based on the variables of alcoholic drinks (X16), energy drinks (X14), soft/carbonated drinks (X13), mental-emotional disorders (X17), Fruit and Vegetables Consumptions (X21), diagnosed hypertension (X18), Physical Activity (X19), and Marital Status (X2).

In addition to the Logistic Regression method, prediction of obesity classification also used CART and Naïve Bayes methods. From Figure 4, it can be seen that the characteristics of the variables that influence the occurrence of obesity in the Indonesia RISKESDAS 2018 are significant variables that function as the main partitioning of all the trees produced. In this case, the main partitioning variables for 10% test data with fold 6 out of the 10-fold CV are alcoholic drinks (X16). The order of important variables in this CART model are alcoholic drinks (X16), energy drinks (X14), soft/carbonated drinks (X13), mental-emotional disorders (X17), fruit and vegetables consumptions (X21), diagnosed hypertension (X18), physical activity (X19), and marital status (X2).

Obesity prediction using the Naïve Bayes model was also done by looking for values of *P*(*C*_*i*_) for the obese class and *P*(*C*_*j*_)for the non-obese class. In this case, the value of *i* = 1 and the value of *j* = 2. The probability value for each variable on the class label is presented in detail in the Supplementary Table 3.

## Discussion

We have conducted a study to establish a set of risk factors for obesity in adults among the available study variables using ML methods using publicly available data on RISKESDAS (RISKESDAS 2018). In this study, three methods (Logistic Regression, CART, and Naïve Bayes) were used in the ML approach to select a method that produces predictions with high accuracy. The result revealed that the Logistic Regression method shows a better accuracy compared to the other methods with AUC = 0.798 using 21 variables, namely location (X1), marital status (X2), age groups (X3), education (X4), work types (X5), sugary foods (X6), sweet drinks (X7), fatty/oily foods (X9), grilled foods (X10), preserved foods (X11), seasoning powders (X12), soft/carbonated drinks (X13), energy drinks (X14), instant foods (X15), alcoholic drinks (X16), mental emotional disorders (X17), diagnosed hypertension (X18), physical activity (X19), smoking (X20), and fruit and vegetables consumptions (X21).

With the accelerated economic growth and lifestyle changes around the world, including in Indonesia, it is important to evaluate and build predictive models for obesity using common risk factors. Based on RISKESDAS 2013 and 2018, Indonesia as a middle-income country seems to underestimate the significance of actual obesity cases even though there has been a significant increase in cases. As shown in this study, the 21 selected measures play a prominent role in increasing the risk for obesity in adults. This is in parallel with some previous studies. In their study, Roemling and Qaim (4) found that obesity risk in Indonesia occurred both in rural and urban areas and was closely associated with food consumption pattern changes coupled with physical activity decreases. Rachmi et al. (5) showed that the increasing prevalence of overweight children, adolescents, and adults in Indonesia over the past two decades coincides with higher numbers of obesity in urban areas. Similarly, Oddo et al. (6) demonstrated that there were more obesity cases in rural areas compared to the past even though the overall case numbers are still higher in urban areas in Indonesia. They also showed that highly processed foods are mostly consumed and decreased physical activities have led to the higher prevalence of obesity. Dewi et al. (7) found that the consumption of oil and fat, animal source foods, and low physical activities are some of the significant determinants of obesity in Indonesia. Emery et al. (40) revealed that there was a relationship between less healthy food consumption with obesity. Sinha and Jastreboff (41) found that eating habits and the increased consumption of food result from stress. Koski and Naukkarinen (42) strengthened the fact that the development of obesity is significantly due to persistent stress. The difference in confounding factors involved in the analysis is one of the reasons for the differences found in this study with previous studies.

In this study, we employed the metrics for accuracy, sensitivity, specificity, precision, recall, F1-score, kappa, and *F*_β_ with 10-fold CV for performance evaluation of the three classification methods. The results obtained are the prediction of the classification with 10-fold CV using the Logistic Regression method, which achieved the best performance as assessed by the accuracy metric (72%), specificity (71%), precision (69%), kappa (44%), and *F*_β = 0.5_ (70%). Classification prediction by the CART method achieved the highest sensitivity (82%), and F1-score (72%). The Naïve Bayes method had an accuracy of 71% and a *F*_β = 0.5_ of 69%.

In general, this ML approach is an alternative to the classical methods used so far (22). Using ML methods on public health data can help to improve predictions and find a rich structure among available data and increase understanding of complex problems in public health, including risk factors for obesity with ML. The ML method could inform the design of more appropriate health policies and programs to address Non-Communicable Diseases, most notably in predicting obesity incidence/prevalence, and in turn, reducing severity as well as the cost of treating obesity and obesity-related condition which eventually could improve the health and well-being of the population. Apart from that, the ML method as shown in the current study could be utilized to identify the most significant risk factors for predicting obesity status can be applied to publicly available data, such as RISKESDAS data.

In general, RISKESDAS provides an overview of Indonesian health indicators, such as health status, health services, health behavior, and environmental health. RISKESDAS is supposedly the best data available on health in Indonesia but its main limitation is the fact that the purpose and nature of RISKESDAS are based on a periodic study (every 5 years) examining a broad range of health issues and health behaviors. This then results in a data set that lacks depth.

In Indonesia, policies on obesity prevention and control in adults are related to limiting consumption of fats and oils, sugary foods and carbohydrates, and increasing vegetable intake are carried out through the Health Community Movement, known as GERMAS and the Food Label with the inclusion of sugar, salt, and fat content on food labels (7). Yet, these efforts seem to be ineffective as the increase in the proportion of obesity remains relatively high. The findings of this study in predicting the risk factor for obesity among the available study variables on RISKESDAS 2018 can then convince the policy makers in Indonesia (primarily the government) to put more attention into the pressing obesity problems. As a result, the effectiveness of existing program policies could be further improved and the financing of the health care system can be made more efficient (43).

This study provides an overview of the methods available for predicting risk factors for obesity in adults among the available study variables in Indonesia. Several factors that might influence obesity (e.g., sex, dietary quality, clinical and physiological, wealth, genetic and cultural influences) were not included in this study, and thereby, the relationship between these factors and obesity cannot be explained further. Further research needs to be carried out using large datasets with individual subjects to confirm the results of this study and to describe the variation in the results for individual regions.

## Conclusion

The Logistic Regression method showed better results on the accuracy, specificity, precision, kappa, and *F*_β_ metrics. Meanwhile, the CART method showed better results on the sensitivity, recall, and F1-score. For the 10-fold CV, the Logistic Regression method had the highest AUC performance which was 0.798. Then, from the Logistic Regression method, it can also be seen that the variables that affect the prediction of obesity status in adults are location, marital status, age groups, education, sweet drinks, fatty/oily foods, grilled foods, preserved foods, seasoning powders, soft/carbonated drinks, alcoholic drinks, mental emotional disorders, diagnosed hypertension, physical activity, smoking, and fruit and vegetables consumptions. The constructed obesity classification model can evaluate and predict the risk of obesity using ML methods for the population of Indonesia which can then be applied to publicly available open data, such as the RISKESDAS survey data. In general, this study has been able to establish a set of risk factors for obesity in adults among the available study variables. However, more studies should be done to further improve the quality of predictions by exploring other ML models. In the future work, we will validate the results with other relevant groups. Additionally, we will also evaluate differences in the prediction of obesity status at the district/city or province level in Indonesia with regional disaggregation.

## Data Availability Statement

Publicly available datasets were analyzed in this study. This data can be found at: https://www.litbang.kemkes.go.id/layanan-permintaan-data-riset.

## Author Contributions

ST contributed to the concept and design of the study, carried out the statistical analysis, and wrote the manuscript. DA interpreted the data, analyzed, and wrote the manuscript. HK collected the necessary data and carried out the statistical analysis. AL interpreted the data and analyzed the manuscript. SN analyzed and wrote the manuscript. All authors read and approved the final manuscript.

## Conflict of Interest

The authors declare that the research was conducted in the absence of any commercial or financial relationships that could be construed as a potential conflict of interest.
