# Supplementary material for: Predicting Obesity in Adults Using Machine Learning Techniques: An Analysis of Indonesian Basic Health Research 2018
Source: Front Nutr. 2021 Jun 21;8:669155. doi: 10.3389/fnut.2021.669155 (PMC8255629; doi:10.3389/fnut.2021.669155)
Supplement: Supplementary file 1 [file Data_Sheet_1.pdf]

frontiers

Supplementary Material

1 Supplementary Tables

Table S1. Description of obesity dataset from Indonesian RISKESDAS Survey 2018.

	Variables	Categories	Composite/Calculation
	Outcome		
1	Obesity Status	1. Obese	Derived from BMI Calculation for Adult (>=18 years) according WHO Standard:<br>Weight (kg)/ Height^2 (m)
		2. Non-Obese	Obese: >=30; Non-Obese <30
	Predictors		
2	Location	1. Urban	According to Indonesia Central Bureau of Statistics (BPS) Regulation (Perka BPS No. 37 Tahun 2010)
		2. Rural	
3	Sex	1. Male	
		2. Female	
4	Marital Status	1. Not Married	
		2. Married	
		3. Divorced	
		4. Widowed	
5	Age group	18-20 Years	
		20 - 24 Years	
		25 - 29 Years	
		30 - 34 Years	
		35 - 39 Years	
		40 - 44 Years	
		45 - 49 Years	
		50 - 54 Years	
		55 - 59 Years	
		60 - 64 Years	
		> 64 Years	
6	Education	Not Schooled	
		Not finished basic school	
		Finished basic school	
		Finished Junior High School	

		Finished Senior High School
		Finished Academy/College
		Finished Higher Education
7	Occupation	Not Working
		School
		Government Employee
		Private Employee
		Entrepreneur
		Farmer
		Fisherman
		Daily Waged Labours
		Others
8	Ever Diagnosed Hypertension	Yes
		No
		Unknown
9	Sugary Foods	> 1 time per day
		1 time per day
		3-6 times per week
		1 -2 times per week
		< 3 times per month
		Never
10	Sweet Drinks	> 1 time per day
		1 time per day
		3-6 times per week
		1 -2 times per week
		< 3 times per month
		Never
11	Salty Foods	> 1 time per day
		1 time per day
		3-6 times per week
		1 -2 times per week
		< 3 times per month
		Never
12	Fatty/Oily Foods	> 1 time per day
		1 time per day
		3-6 times per week
		1 -2 times per week
		< 3 times per month
		Never
13	Grilled Foods	> 1 time per day
		1 time per day

		3-6 times per week<br>1 -2 times per week<br>< 3 times per month<br>Never	
14	Preserved Foods	> 1 time per day<br>1 time per day<br>3-6 times per week<br>1 -2 times per week<br>< 3 times per month<br>Never	
15	Seasonings Powders	> 1 time per day<br>1 time per day<br>3-6 times per week<br>1 -2 times per week<br>< 3 times per month<br>Never	
16	Soft Drinks	> 1 time per day<br>1 time per day<br>3-6 times per week<br>1 -2 times per week<br>< 3 times per month<br>Never	
17	Energy Drinks	1 time per day<br>3-6 times per week<br>1 -2 times per week<br>< 3 times per month<br>Never	
18	Instant Foods	1 time per day<br>3-6 times per week<br>1 -2 times per week<br>< 3 times per month<br>Never	
19	Fruit and Vegetable consumptions	Adequate<br>Not Adequate	Using WHO STEPWISE Instrument for Fruit and Vegetable Consumption with Criteria:<br>Adequate: 5 portions of Fruit and Vegetables / day
20	Physical Activity	Adequate<br>Not Adequate	Using GPAQ Instrument and Standard to measure physical activity adequacy
21	Smoking Status	Yes<br>No	Considered as smoking if currently or recently quit smoking

22	Alcoholic Drinks	Yes<br><br><br>No	Using WHO STEPWISE Instrument for Alcohol Consumption<br>Yes: >=5 Standard volume (male) or >=4 Standard volume for female
23	Mental/Emotional Disorders	Yes<br><br><br>No	Using Self Reporting Questionnaire - 20 (SRQ-20) WHO.<br>Score of >=8 Considered as Having mental/ emotional disorders

Table S2. Description of obesity status from Indonesian RISKESDAS Survey 2018

Variables	Categories	Obesity Status		Total
		Non-obese	Obese	
Location (X1)	Urban	197,456	70,457	267,913
	Rural	286,733	64,252	350,985
Marital Status (X2)	Not married	75,449	9,343	84,792
	Married	359,116	113,153	472,269
	Divorced	11,400	2,933	14,333
	Widowed	38,224	9,280	47,504
Age Groups (X3)	18 - 24 Years	61,926	7,606	69,532
	25 - 29 Years	49,499	10,881	60,380
	30 - 34 Years	53,175	15,508	68,683
	35 - 39 Years	57,094	20,444	77,538
	40 - 44 Years	52,549	21,226	73,775
	45 - 49 Years	50,726	19,777	70,503
	50 - 54 Years	43,613	15,005	58,618
	55 - 59 Years	38,598	11,034	49,632
	60 - 64 Years	28,679	6,792	35,471
	> 64 Years	48,330	6,436	54,766
Education (X4)	Not/Never Schooled	35,258	5,603	40,861
	Not finished basic school	69,572	15,065	84,637
	Finished basic school	124,463	32,928	157,391
	Finished Junior High School	80,376	24,059	104,435
	Finished Senior High School	130,912	39,334	170,246

	Finished Academy/College	14,532	5,473	20,005
	Finished Higher Education	29,076	12,247	41,323
Work Types (X5)	Not Working	125,504	46,480	171,984
	School	10,703	1,535	12,238
	Government Employee	18,100	9,603	27,703
	Private Employee	38,945	11,104	50,049
	Entrepreneur	66,585	24,426	91,011
	Farmer	141,901	21,108	163,009
	Fisherman	7,282	1,062	8,344
	Daily Waged Labours	43,792	8,587	52,379
	Others	31,377	10,804	42,181
Sugary Foods (X6)	>1 time per day	64,728	18,047	82,775
	1 time per day	97,588	28,166	125,754
	3-6 times per week	109,134	29,551	138,685
	1 -2 times per week	138,537	38,636	177,173
	< 3 times per month	49,293	13,679	62,972
	Never	24,909	6,630	31,539
Sweet Drinks (X7)	>1 time per day	143,500	32,596	176,096
	1 time per day	152,266	43,095	195,361
	3-6 times per week	68,279	19,548	87,827
	1 -2 times per week	72,677	22,732	95,409
	< 3 times per month	25,036	8,630	33,666
	Never	22,431	8,108	30,539
Salty Foods (X8)	>1 time per day	50,731	13,929	64,660
	1 time per day	61,411	17,333	78,744
	3-6 times per week	82,878	22,485	105,363
	1 -2 times per week	134,363	36,079	170,442
	< 3 times per month	83,436	23,882	107,318
	Never	71,370	21,001	92,371
Fatty/Oily Foods (X9)	>1 time per day	79,104	24,530	103,634
	1 time per day	86,353	26,704	113,057
	3-6 times per week	104,265	29,287	133,552
	1 -2 times per week	129,898	34,805	164,703
	< 3 times per month	58,867	13,872	72,739
	Never	25,702	5,511	31,213
Grilled Foods (X10)	>1 time per day	10,318	2,630	12,948
	1 time per day	17,294	4,895	22,189
	3-6 times per week	49,777	14,190	63,967
	1 -2 times per week	124,605	36,751	161,356
	< 3 times per month	157,720	44,531	202,251

	Never	124,475	31,712	156,187
Preserved Foods (X11)	>1 time per day	4,815	1,495	6,310
	1 time per day	9,142	2,882	12,024
	3-6 times per week	24,801	7,192	31,993
	1 -2 times per week	55,947	16,671	72,618
	< 3 times per month	113,973	31,095	145,068
	Never	275,511	75,374	350,885
Seasonings Powders (X12)	>1 time per day	179,277	48,080	227,357
	1 time per day	176,181	50,447	226,628
	3-6 times per week	33,493	9,105	42,598
	1 -2 times per week	26,880	7,150	34,030
	< 3 times per month	16,460	4,427	20,887
	Never	51,898	15,500	67,398
Soft / Carbonated Drinks (X13)	>1 time per day	2,975	714	3,689
	1 time per day	6,288	1,569	7,857
	3-6 times per week	13,311	3,159	16,470
	1 -2 times per week	35,336	8,350	43,686
	< 3 times per month	79,357	21,041	100,398
	Never	346,922	99,876	446,798
Energy Drinks (X14)	>1 time per day	3,039	615	3,654
	1 time per day	6,372	1,389	7,761
	3-6 times per week	10,926	1,962	12,888
	1 -2 times per week	26,092	4,953	31,045
	< 3 times per month	48,399	10,260	58,659
	Never	389,361	115,530	504,891
Instant Foods (X15)	>1 time per day	9,725	2,419	12,144
	1 time per day	23,167	5,776	28,943
	3-6 times per week	86,552	21,735	108,287
	1 -2 times per week	172,695	47,430	220,125
	< 3 times per month	114,255	34,811	149,066
	Never	77,795	22,538	100,333
Alcoholic Drinks (X16)	Yes	26,023	4,217	30,240
	No	458,166	130,492	588,658
Mental-Emotional Disorders (X17)	Yes	48,336	12,756	61,092
	No	435,853	121,953	557,806
Diagnosed Hypertension (X18)	Yes	35,895	19,745	55,640
	No	240,745	74,722	315,467
	Unknown	207,549	40,242	247,791
Physical Activity (X19)	Adequate	61,465	12,271	73,736
	Not Adequate	422,724	122,438	545,162
Smoking (X20)	Yes	201,708	31,598	233,306
	No	282,481	103,111	385,592
	Adequate	21,543	7,778	29,321

Fruit and Vegetables Consumptions (X21)	Not Adequate	462,646	126,931	589,577

Table S3. Probability of each variable on the label of obesity data class using Naïve Bayes method based on fold 6 out of 10-fold CV

Variables	Categories	Fold 6 out of 10-fold cross validation Test	
		Non-obese	Obese
Location (X1)	Urban	0.408	0.507
	Rural	0.592	0.493
Marital Status (X2)	Not married	0.156	0.219
	Married	0.742	0.624
	Divorced	0.024	0.056
	Widowed	0.079	0.101
Age Groups (X3)	18 - 24 Years	0.128	0.108
	25 - 29 Years	0.102	0.120
	30 - 34 Years	0.110	0.129
	35 - 39 Years	0.118	0.138
	40 - 44 Years	0.109	0.133
	45 - 49 Years	0.105	0.131
	50 - 54 Years	0.090	0.090
	55 - 59 Years	0.080	0.063
	60 - 64 Years	0.059	0.048
	> 64 Years	0.100	0.039
Education (X4)	Not/Never Schooled	0.073	0.039
	Not finished basic school	0.144	0.120
	Finished basic school	0.257	0.223
	Finished Junior High School	0.166	0.176
	Finished Senior High School	0.270	0.324
	Finished Academy/College	0.030	0.037
	Finished Higher Education	0.060	0.082
Work Types (X5)	Not Working	0.259	0.259
	School	0.022	0.020
	Government Employee	0.037	0.064
	Private Employee	0.080	0.103
	Entrepreneur	0.138	0.187
	Farmer	0.293	0.198
	Fisherman	0.015	0.009
	Daily Waged Labours	0.091	0.084
	Others	0.065	0.076
Sugary Foods (X6)	>1 times per day	0.134	0.169

	1 times per day	0.201	0.204
	3-6 times per week	0.225	0.215
	1-2 times per week	0.286	0.252
	< 3 times per month	0.102	0.113
	Never	0.051	0.048
Sweet Drinks (X7)	>1 times per day	0.297	0.284
	1 times per day	0.314	0.305
	3-6 times per week	0.141	0.147
	1-2 times per week	0.150	0.149
	< 3 times per month	0.052	0.065
	Never	0.046	0.050
Salty Foods (X8)	>1 times per day	0.105	0.118
	1 times per day	0.127	0.152
	3-6 times per week	0.171	0.163
	1-2 times per week	0.277	0.248
	< 3 times per month	0.172	0.171
	Never	0.147	0.147
Fatty/Oily Foods (X9)	>1 times per day	0.163	0.190
	1 times per day	0.178	0.191
	3-6 times per week	0.216	0.213
	1-2 times per week	0.268	0.256
	< 3 times per month	0.121	0.117
	Never	0.053	0.033
Grilled Foods (X10)	>1 times per day	0.021	0.043
	1 times per day	0.036	0.059
	3-6 times per week	0.103	0.138
	1-2 times per week	0.257	0.264
	< 3 times per month	0.326	0.304
	Never	0.257	0.191
Preserved Foods (X11)	>1 times per day	0.010	0.033
	1 times per day	0.019	0.034
	3-6 times per week	0.051	0.076
	1-2 times per week	0.116	0.153
	< 3 times per month	0.236	0.255
	Never	0.569	0.449
Seasonings Powders (X12)	>1 times per day	0.371	0.324
	1 times per day	0.364	0.338
	3-6 times per week	0.069	0.088
	1-2 times per week	0.056	0.064
	< 3 times per month	0.034	0.056
	Never	0.107	0.129
Soft / Carbonated Drinks (X13)	>1 times per day	0.006	0.012
	1 times per day	0.013	0.041

	3-6 times per week	0.027	0.063
	1-2 times per week	0.073	0.138
	< 3 times per month	0.164	0.228
	Never	0.716	0.518
Energy Drinks (X14)	>1 times per day	0.006	0.015
	1 times per day	0.013	0.041
	3-6 times per week	0.022	0.054
	1-2 times per week	0.054	0.115
	< 3 times per month	0.100	0.181
	Never	0.804	0.594
Instant Foods (X15)	>1 times per day	0.020	0.028
	1 times per day	0.048	0.062
	3-6 times per week	0.179	0.206
	1-2 times per week	0.357	0.323
	< 3 times per month	0.236	0.244
	Never	0.161	0.137
Alcoholic Drinks (X16)	Yes	0.054	0.254
	No	0.946	0.746
Stress (X17)	Yes	0.100	0.268
	No	0.900	0.732
Diagnosed Hypertension (X18)	Yes	0.074	0.155
	No	0.497	0.468
	Unknown	0.429	0.377
Physical Activity (X19)	Adequate	0.127	0.250
	Not Adequate	0.873	0.750
Smoking (X20)	Yes	0.417	0.434
	No	0.583	0.566
Fruit and Vegetables Consumptions (X21)	Adequate	0.045	0.162
	Not Adequate	0.955	0.838
